# LNC_000280 could be a new positive factor in the proliferation and differentiation of myoblasts: A prospective study

**DOI:** 10.1371/journal.pone.0313679

**Published:** 2024-11-27

**Authors:** Shen Wang, Xinyi Gu, Qinghe Geng, Jin Deng, Chen Huang, Shuhang Guo, Qingguo Lu, Xiaofeng Yin

**Affiliations:** 1 Department of Orthopedics and Traumatology, Peking University People’s Hospital, Beijing, China; 2 Key Laboratory of Trauma and Neural Regeneration (Peking University), Beijing, China; 3 Pizhou People’s Hospital, Xuzhou, Jiangsu Province, China; Yeungnam University, REPUBLIC OF KOREA

## Abstract

Peripheral nerve injury may result in muscle atrophy and impaired motor function recovery, and numerous pieces of evidence indicate that long noncoding RNAs (lncRNAs) play crucial roles in skeletal muscle regeneration. Our preliminary sequencing results showed that LNC_000280 was significantly down-regulated in denervated mouse skeletal muscle and we hypothesized that LNC_000280 may play an important role in skeletal muscle regeneration. In this research, flow cytometry and EdU staining results showed that overexpression of LNC_000280 promoted the proliferation of C2C12, while knockdown LNC_000280 had the opposite effect. Knockdown LNC_000280 inhibited the differentiation of C2C12 cells. LNC_000280 regulated the expression of proliferation genes (*Cdk2*, *Cdc27*) and differentiation genes (*MyoG*, *MyoD*). GO analysis and PPI network of LNC_000280 target genes showed that LNC_000280 mainly regulates skeletal muscle cell metabolism, mitochondrial and muscle growth. *Idh2*, *Klhl31*, *Agt*, and *Gpt2* may be important downstream targets for its function. Therefore, we believe that that LNC_000280 can regulate the proliferation and differentiation of myoblasts by regulating gene expression.

## Introduction

Peripheral nerve injury can lead to motor function degradation and disruption of blood networks, causing a series of pathological reactions, including oxidative stress, inflammation, mitochondrial damage, and loss of protein and muscle structure. As the absence of innervation, collagen will build up in muscle fibers, exacerbating skeletal muscle atrophy by impeding cellular signal transduction [1–5].

Long non-coding RNAs (lncRNAs) are a type of functional RNA composed of over 200 bases, which cannot express proteins but can regulate gene expression [6, 7]. lncRNAs can be involved in regulation at multiple stages of gene expression, and there has been a lot of evidence identifying them as important regulators of muscle regeneration [8, 9]. For instance, LncFAM can promote muscle production by regulating *MYBPC2* expression [10]. According to our previous results [11, 12], we found that the expression of LNC000280 was significantly downregulated in skeletal muscles with neuroatrophy and have validated the modulatory effect of LNC_000280 on acetylcholine receptors in C2C12 [12].

Could LNC_000280 overexpression or knockdown result in deeper functional changes in myoblasts? Based on this, we designed a series of experiments to verify whether LNC_000280 would have an impact on proliferation or differentiation. Over the past few years, evidence from numerous studies suggests that cell division cycle 27(*Cdc27*) and cyclin-dependent kinase 2 (*Cdk2*) has a positive effect of skeletal muscle cell proliferation [13–17]. Otherwise, there have been numerous studies showed that members of myogenic regulatory factors (*MRFs*) such as MyoD and MyoG work together to activate gene expression and promote the formation of myoblasts [18–20]. Besides, in skeletal muscle, increased *MyoG* expression will mean the preparation for differentiation and exits the cell cycle irreversibly and generally considered to be a key factor in end-stage cell differentiation. Here, we will detect the mRNA expressions of aforementioned to observe whether LNC_000280 as an important factor on myoblast proliferation and differentiation.

In this study, we utilized a lentivirus packaging plasmid to establish four groups: LNC_000280 overexpression, overexpression control, knockdown, and knockdown control. A series of related experiments were conducted to verify the role of LNC_000280 in regulating myofibroblast function. Upon comparing the results of each group and analyzing the expression of *Cdk2*, *Cdc27*, *MyoD* and *MyoG*, we hypothesize that LNC_000280 significantly affects the proliferation and differentiation of C2C12 cells, which is supported by the PPI network and GO enrichment analysis of its target genes.

## Materials and methods

### Lentiviral vector construction and cell transfection

We used lentiviral packaging plasmids to establish the overexpression group, knockdown group, and their respective negative control groups (hU6-MCS-Ubiquitin-EGFP-IRES-puromycin, Genechem).

We seeded and cultured the myoblasts until they reached 30% confluence. The medium was removed and viral particles were introduced (HiTransG A, Genechem). Cells expressing green fluorescence under a fluorescence microscope were considered successfully transfected (Zeiss Axio Vert A1, Germany). The transfection efficiency was determined as the ratio of cells expressing green fluorescent protein to the total cell count.

### Gene expression level detection

RNA-Quick Purification Kit (ESscience Biotech, Shanghai, China) was used to extract total RNA from the myotubes differentiated for three days according to the manufacturer’s instructions.

The q-PCR primers of microRNAs were as follows:

LNC_000280-F: 5’-CAAGGAATGCAGCTTTAGAC-3’

LNC_000280-R: 5’-GGATTTGCTACAGTCAGGGT-3’

*Cdk2*-F: 5’-ATGGAGAACTTCCAAAAGGTGG-3’

*Cdk2*-R: 5’-CAGTCTCAGTGTCGAGCCG-3’

*Cdc27*-F: 5’-TCCAGGTCGATCCAAATTACGCT-3’

*Cdc27*-R: 5’-AGGCGAGATGAGTTTGACCCA-3’

*MyoD*-F: 5’-CATTCCAACCCACAGAACCT-3’;

*MyoD*-R: 5’-CAAGCCCTGAGAGTCGTCTT-3’;

*MyoG*-F: 5’-CAATGCACTGGAGTTCGGT-3’;

*MyoG*-R: 5’-CTGGGAAGGCAACAGACAT-3’.

### EdU assays

Myoblasts were transfected for 24 or 36 hours and then incubated with 50 mM EdU for 3 hours (EdU Assay Kit, Ribobio, C10310-2). After the addition of EdU, the process involves sequentially fixing (4% paraformaldehyde), permeabilizing (0.5% Triton X-100), incubating (Apollo Reaction), and staining (Hoechst 33342) the cells.

### Cell cycle flow cytometry

Cells were fixed overnight -20°C, 70% ethanol) after transfection for 24 or 36 hours. Subsequently, the cells were incubated with the prepared solution (including 50 μg/mL propidium iodide, 100 μg/mL RNase A, 0.2% Triton X-100).

### Immunofluorescence

After fixing the cells (4% paraformaldehyde), they were washed three times with PBS, 5 minutes each time. The cells were then permeabilized (0.1% Triton X-100, 15min) and incubated (Beyotime, 1h), followed by incubation at 4°C with anti-*MHC* antibody overnight (M4276, Sigma). The next day, incubate the sample with the second antibody (Alexa Fluor 594-conjugated goat anti-mouse IgG, Invitrogen, 2h). Cell nuclei were visualized with DAPI staining (Roche).

### Prediction of LNC_000280 target genes

The co-expressed genes of LNC_000280 were obtained based on the published sequencing results (PMID: 29563865). The Pearson correlation coefficients between genes and LNC_000280 were calculated based on the gene expression data to construct the expression regulatory network. The criteria for gene inclusion are p < 0.05 and | r | > 0.95. The complete sequence information and co-expressed genes of LNC_000280 can be found in S1 File.

### Gene Ontology (GO) enrichment analysis

We utilized DAVID (https://david.ncifcrf.gov/) to analyze the genes that were co-expressed. Excel was used for graphing, displaying only pathways associated with skeletal muscle proliferation and differentiation.

### Construction of the Protein-Protein Interaction network (PPI)

The co-expressed genes were imported into String v.11.5 to get the PPI raw data. Imported the data into Cytoscape_3.7.2 for graphing, the higher the degree of the nodes closer to the center in the graph. Proteins known to influence skeletal muscle proliferation and differentiation were then labeled.

### Protein interaction diagram constructed by Genemania

Genemania (https://genemania.org/) was used to calculate the interaction relationship between LNC_000280 target genes and *Cdk2*, *Cdc27*, *MyoG*, and *MyoD*. Drawing completed using Microsoft Office PowerPoint 2019.

### Statistical analysis

Data analysis and graphing were performed using Prism v8.2.1 and OriginPro 2019, Student’s t-test. And all figure showed the mean ± standard error. All statistical data can be found in S2 File.

## Results

### 1. LNC_000280 overexpression and knockdown cell model

**Fig 1:**
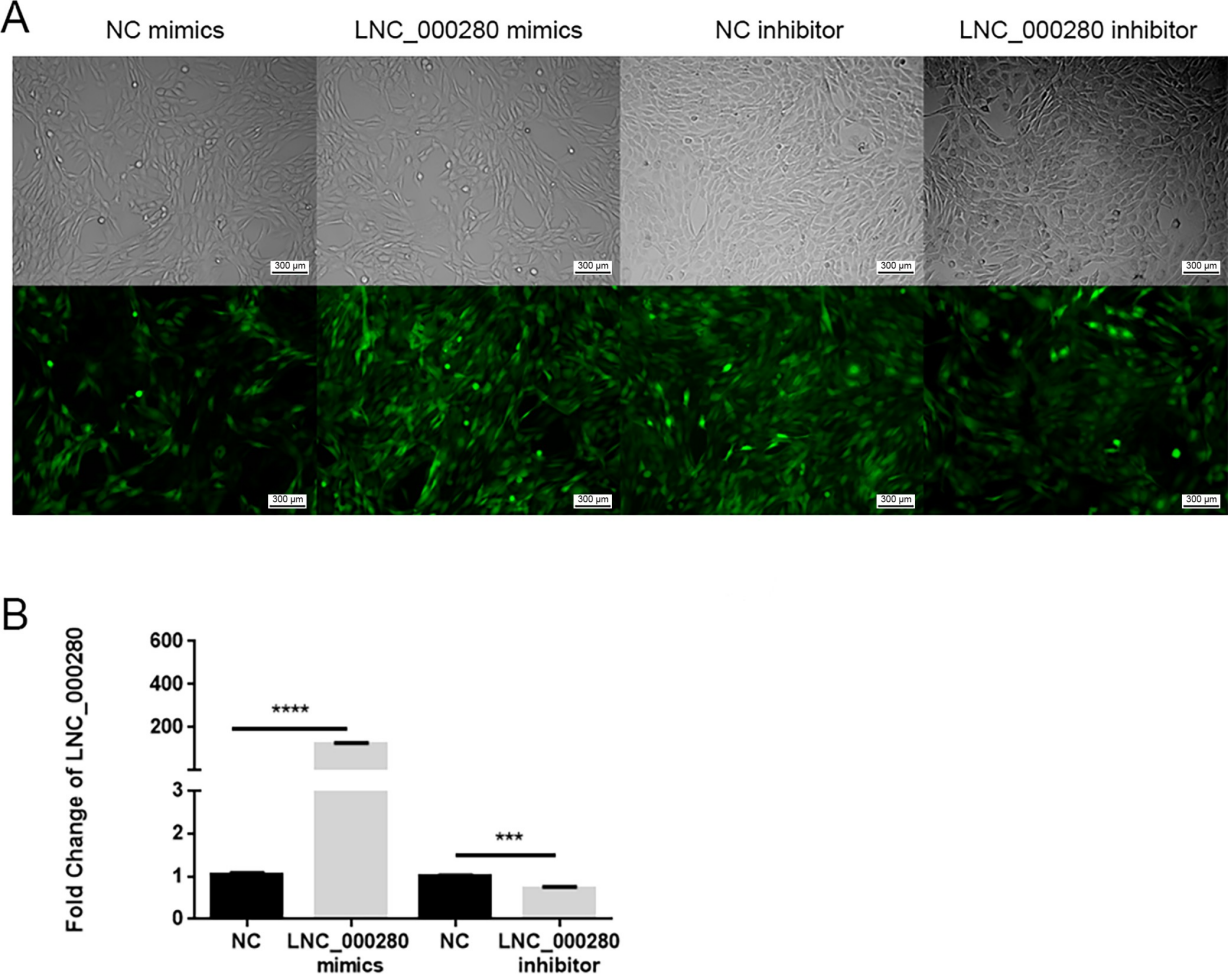
LNC_000280 overexpression and knockdown cell model. (A) Fluorescence microscope observed that the plasmid stably expressed GFP tag-protein in each group. (n = 6, scale bar, 300 μm). (B) qRT-PCR results showed the expression of LNC_000280. Data presented as mean ± SD. *p<0.01.

In this study, we constructed LNC_000280 knockdown and overexpression cells by transfecting C2C12 with plasmid packaged with lentivirus. The plasmid carries a green fluorescent label, so observing the proportion of C2C12 expressing green fluorescence under a fluorescence microscope can determine the transfection efficiency of the plasmid. We observed that most cells expressed green fluorescence and confirmed the successful construction of the cell model through PCR (Fig 1A, 1B).

### 2. LNC_000280 promotes the proliferation of C2C12

**Fig 2:**
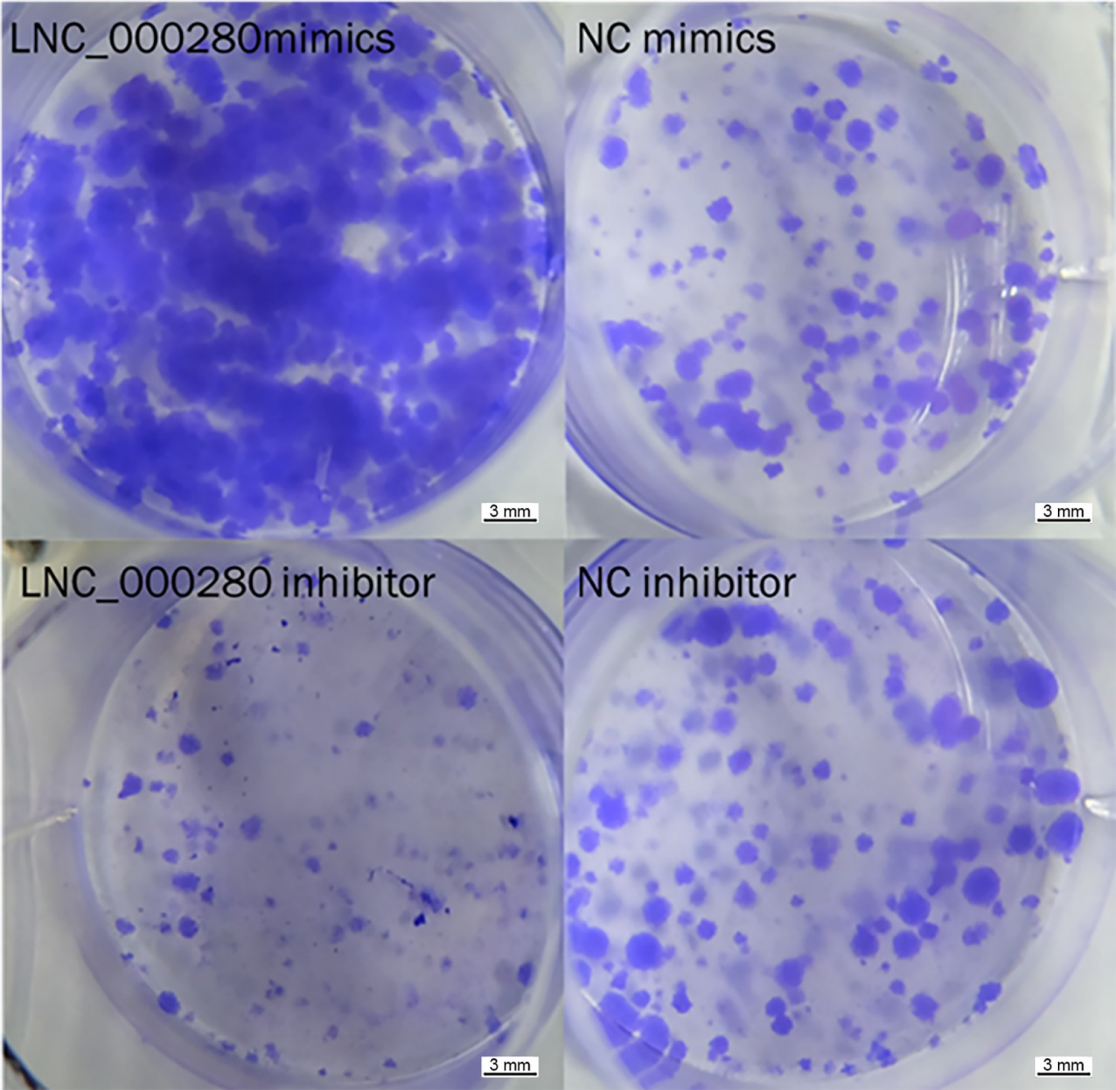
LNC_000280 promotes the proliferation of C2C12. Plate cloning experiment showed that overexpression of LNC_000280 could significantly promote cell proliferation. n = 3, Scale bar = 3 mm.

**Fig 3:**
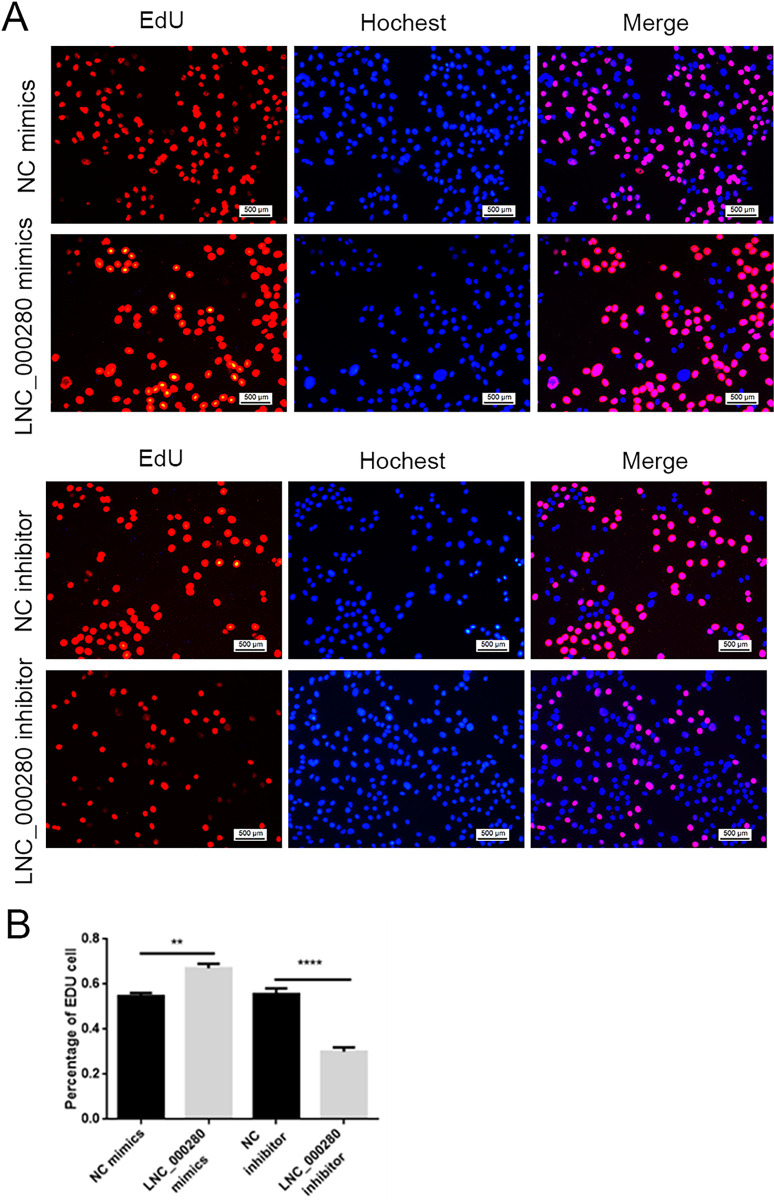
LNC_000280 increasing the proportion of C2C12 cells in S phase. (A) EdU staining results show that LNC_000280 increases EdU positive cells (Scale bar, 500 μm). (B)The ratio of overexpression/knockdown LNC_000280 cells in each cell cycle. n = 3, data presented as mean ± SD.*p<0.05; **p<0.01.

**Fig 4:**
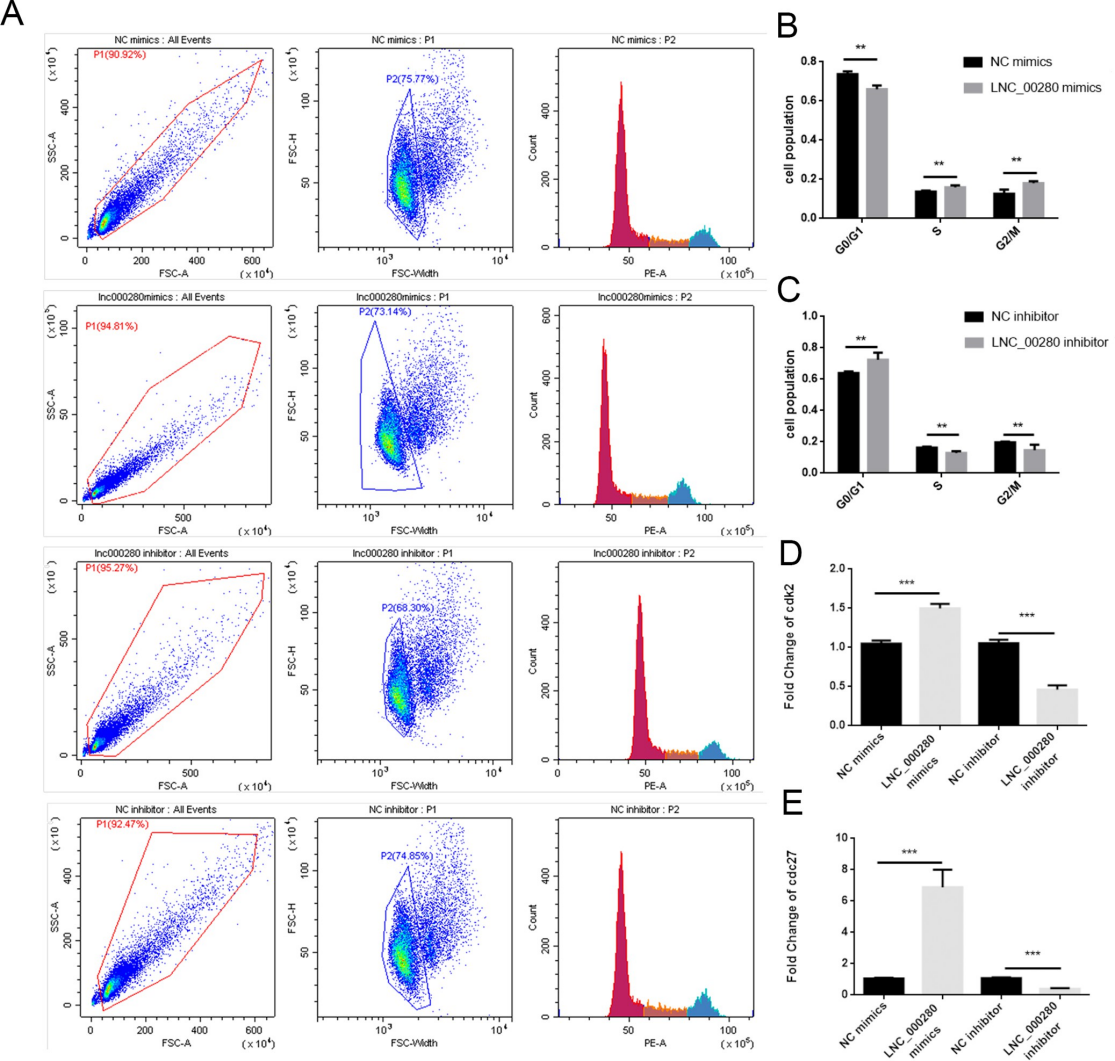
LNC_000280 promotes proliferation of C2C12 and expression of related genes. (A) Cell cycle detection by flow cytometry. (B-C) The overexpression of LNC_000280 increased resulted in an increased number of cells in both S and G2/M phases, while decreasing the number of cells in G0/G1 phase. (D-E) The overexpression of LNC_000280 increased the mRNA expression of *Cdk2* and *Cdc27*. n = 3 per group, data presented as mean ± SD.*p<0.05; **p<0.01.

Plate cloning experiments showed that the cell density in the LNC_000280 overexpression group was significantly higher than that of the control, while the LNC_000280 knockdown group showed the opposite trend (Fig 2). EdU staining showed that the the proliferative-phase cell was significantly increased in the LNC_000280 overexpression group, while it was decreased in the LNC_000280 knockdown group (Fig 3A and 3B). Flow cytometry showed that the S-phase cells was significantly increased in the LNC_000280 overexpression group, while the G0/G1-phase cells was significantly decreased. The trend was opposite in the knockdown group (Fig 4A–4C). The expression levels of cycle-related genes *Cdk2*, *Cdc27* were measured in each group, and It was found that LNC_000280 promotes their expression (Fig 4D and 4E).

### 3. Knockdown LNC_000280 inhibited cell differentiation

**Fig 5:**
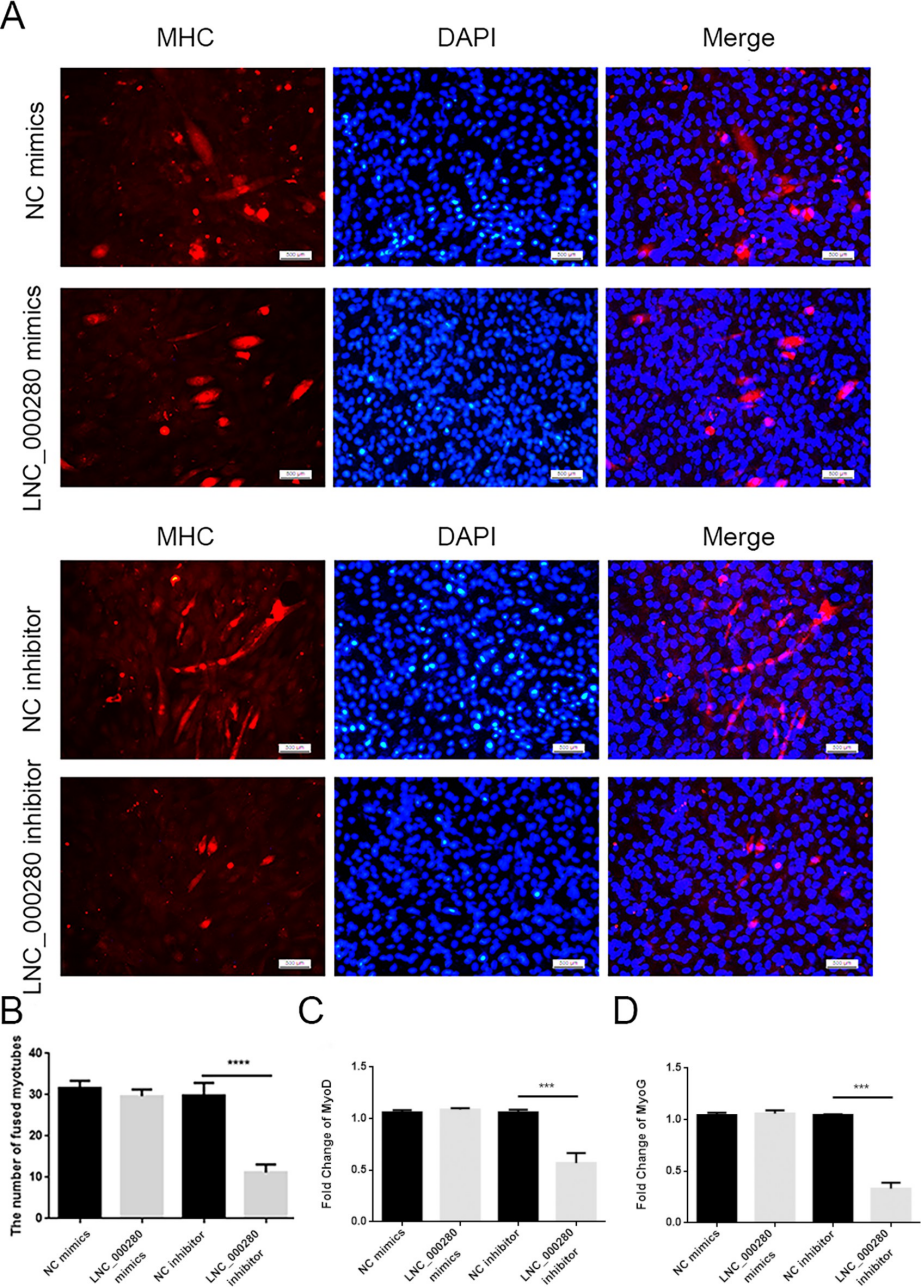
Knockdown LNC_000280 inhibited cell differentiation. (A-B) Immunofluorescence staining, red for *MHC* and blue for DAPI. (C) Count of fused myotubes per field (Scale bar, 500 μm). (d) Knockdown LNC_000280 reduced the expression of *MyoD* and *MyoG*. n = 3, data presented as mean ± SD.*p<0.05; **p<0.01.

Cells in each group were differentiated at the same density for 3 days. The results showed that LNC_000280 knockdown group significantly reduced the number of myotubes, while LNC_000280 overexpression group did not change significantly (Fig 5A and 5B). Meanwhile, LNC_000280 knockdown also significantly inhibited the expressions of *MyoD* and *MyoG* (Fig 5C and 5D).

### 4. Functional analysis of LNC_000280 based on target genes

**Fig 6:**
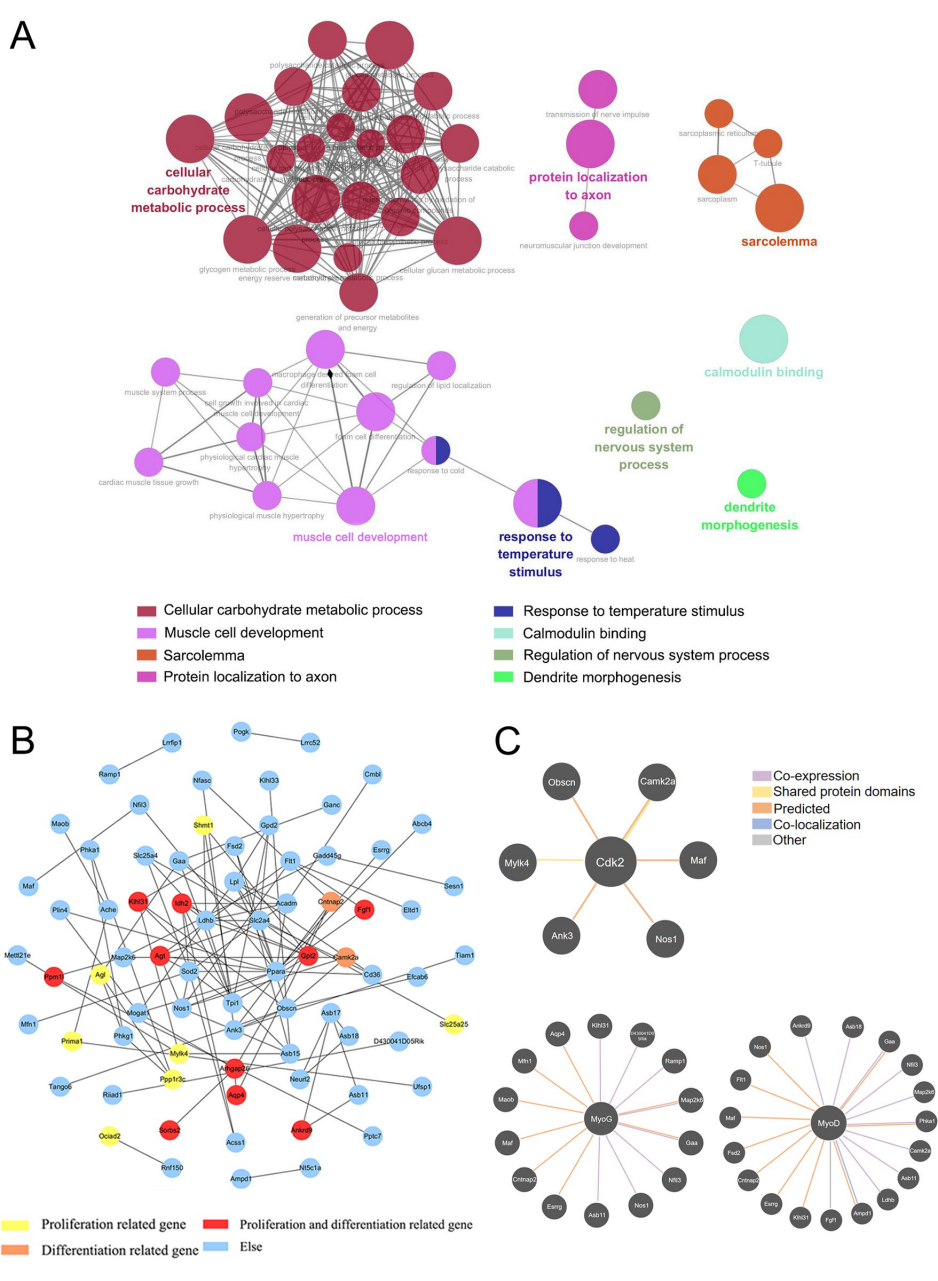
GO enrichment analysis and Protein-protein interaction (PPI) network for LNC_000280 target genes. (A) GO Enrichment Analysis for LNC_000280 target genes (p < 0.05). (B) PPI network of LNC_000280 target genes. The closer the node is to the center, the higher the degree, and the genes related to myoblast function are marked by different colors. (C) The interaction diagram of LNC_000280 target genes with *Cdk2*, *MyoG*, and *MyoD* drawn by GeneMania.

Based on the whole transcriptome sequencing results of denervated muscle published by our group in the past, we calculated the co-expression relationship between LNC_000280 and genes, and obtained the target genes of LNC_000280. We performed GO analysis on these target genes and found that LNC_000280 mainly regulates cell metabolism, muscle growth and protein localization to axon (Fig 6A). PPI (protein interaction network was constructed for the co-expressed genes of LNC_000280. The closer the node is to the center, the higher the degree. The proteins that have been reported to have an effect on skeletal muscle proliferation and differentiation were labeled, and a large number of skeletal muscle proliferation and differentiation-related proteins were found in the PPI (Fig 6B). The PPI results showed that the proliferation-related genes regulated by LNC_000280 included *Shmt1*, *Agl*, *Prima1*, *Mylk4*, *Ppp1r3c*, *Ociad2* and *Slc25a25*; the differentiation-related genes included *Cntnap2* and *Camk2a*; and the genes that regulated proliferation and differentiation included *Idh2*, *Klhl31*, *Agt*, *Gpt2*, *Fgf1*, *Arhgap26*, *Aqp4*, *Ppm1l*, *Sorbs2* and *Ankrd9*. These genes may be important targets for LNC_000280 to regulate myoblast proliferation and differentiation.

To further investigate the mechanism by which LNC_000280 regulates skeletal muscle proliferation and differentiation, we used GeneMania (https://genemania.org/) to explore the interaction between LNC_000280 target genes and *Cdk2*, *Cdc27*, *MyoG*, and *MyoD*, and discovered a group of proteins that interact with *Cdk2*, *MyoG*, and *MyoD* (Table 1, Fig 6C). These proteins are likely important targets for LNC_000280 to regulate C2C12 proliferation and differentiation.

**Table 1:** The interaction relationship between LNC_000280 target genes and *Cdk2*, *MyoG*, and *MyoD*.

	LNC_000280 target genes	Weight	Network group
*Cdk2*	*Camk2a*	0.00527608	Shared protein domains
*Cdk2*	*Mylk4*	0.003553209	Shared protein domains
*Cdk2*	*Ank3*	0.002685871	Predicted
*MyoG*	*Nos1*	0.021587206	Co-expression
*MyoG*	*Gaa*	0.019059548	Co-expression
*MyoG*	*Nfil3*	0.016449029	Co-expression
*MyoD*	*Camk2a*	0.023366265	Co-expression
*MyoD*	*Asb18*	0.022701425	Co-expression
*MyoD*	*Fgf1*	0.017756697	Other

Only show the top three genes with the highest weight.

## Discussion

In our previous research, we conducted transcriptome sequencing analysis on skeletal muscles with neurogenic atrophy, and identified differentially expressed lncRNAs, including LNC_000280 which may be related to the clustering of acetylcholine receptors [12]. As we know, acetylcholine receptors are the important components of the neuromuscular junction and play a key role in muscle function and motor regulation. Therefore, does LNC_000280 have a similar impact on muscle function?

In this study, we discovered that LNC_000280 is related to the proliferation and differentiation of C2C12 cells. In plate cloning experiment, overexpression of LNC_000280 can promote the proliferation of C2C12, and significantly increased the expression of *Cdk2* and *Cdc27*, while knockdown LNC_000280 had the opposite effect. *Cdk2* is a key regulatory factor in the G1 to S phase transition of the cell cycle, mainly regulating the expression and replication of late G1/S phase genes. Meanwhile, *Cdc27* is primarily responsible for promoting the synthesis of complexes/cyclosomes at the late stage of cell cycle [13, 21]. Based on our results, we speculate that overexpression or knockdown of LNC_000280 may affect the number of cells in the G1/S phase transition and thus regulate the process of cell proliferation. In addition, LNC_000280 knockdown significantly inhibited the differentiation process and reduced the formation of myotubes, corresponding to the low expression of *MyoD* and *MyoG*. Myogenic regulatory factors (*MRFs*) are composed of *MyoD*, *MyoG*, *Myf5* and *MRF4*, among which *MyoD* is the first identified myogenic regulatory factor [22]. After muscle injury, satellite cells are activated and express *Myf5* to stimulate myoblast proliferation and then regulate myoblast differentiation during muscle regeneration by expressing *MyoD*. Subsequently, *MyoD* positive cells were withdrawn from the cell cycle and *MyoG* expression was activated to initiate differentiation, this is also reflected in our results that LNC_000280 knockdown may inhibit the differentiation process by influencing the expression of *MyoG* and *MyoD*.

lncRNA extensively participates in various biological processes through its powerful gene expression regulatory ability [23–25]. Through high-throughput technology and bioinformatics analysis, many LncRNAs were found, but only a few were confirmed to be related to muscle regeneration and reconstruction function [26–29]. They promote muscle differentiation and regeneration by regulating related gene expression as competing endogenous RNAs (CERNAs). The previous sequencing results [11] showed that the expression of LNC_000280 was significantly different in the process of denervated muscle atrophy, combined with our experimental results, we believe that LNC_000280 may have the potential to promote the proliferation and differentiation of myoblasts. Furthermore, GO analysis of co-expression genes in LNC_000280 showed a large number of pathways related to skeletal muscle proliferation and differentiation, such as energy reserve metabolic process, multicellular organism development, and cellular metabolic process and so on. PPI network indicated plenty of genes related proliferation and differentiation, *Sorbs2*, *Ankrd9*, *Kihi131*, *Arhgap26* and *Fgf1* have been proven to be closely involved in important muscle biological processes such as proliferation and differentiation [30–36]. These co-expressed genes suggest that the potential of LNC_000280 to influence the proliferation and differentiation of skeletal muscle cells.

In conclusion, our previous research found that LNC_000280 has the ability to regulate the clustering of acetylcholine receptor, this paper further demonstrates LNC_000280 as a new factor could participate in and influence the proliferation and differentiation of myoblasts. Though the mechanism of LNC_000280 was not elaborated, several related pathways and target genes had been provided and would be instructive for intensive study. The limitations of this study include the lack of protein-level detection of cell cycle and differentiation-related proteins. Additionally, the regulatory mechanisms of LNC_000280 were not thoroughly investigated and validated. We plan to further explore these aspects in subsequent studies.

The shortcomings of this article lie in the lack of detection of the protein expression levels of key genes. Due to the interaction between the target gene of LNC_000280 and these key genes (Cdk2, Cdc27, MyoG, and MyoD), we will conduct further investigations into the mechanism of how LNC_000280 affects the dominant expression of C2C12 proliferation and differentiation function through target gene regulation.

## Supporting information

S1 File The full sequence of LNC_000280 and the co-expression genes of LNC_000280. (DOCX)

S2 File Raw data. (XLSX)
