# Supplementary material for: LNC_000280 could be a new positive factor in the proliferation and differentiation of myoblasts: A prospective study
Source: PLoS One. 2024 Nov 27;19(11):e0313679. doi: 10.1371/journal.pone.0313679 (PMC11602059; doi:10.1371/journal.pone.0313679)
Supplement: S1 File — (DOCX) [file pone.0313679.s001.docx]

Supplementary information: S1 DATA

1. The full sequence of LNC_000280

| 5’-CCTGACCTTCTTGTGCTTTTCCAATGAGAAAGTTTAAAGCATGGTCCATAATGACAAGACGCTGCCCACCTGATTGTTTGCAGAGTTGATCCCTTGGGTACCATTGGTCCATCCTGGATACATCACCTAAGCAGAAGATTAAGAAGCTAAGGACTGCCCTCAACTCAGGATTTTTACTCACTGGCTATGAAGCACTCAACTGTGTATTTTGAGCTCCTATTATGATTTGAATGCATGTTGTGCCTCAACATTTCTGTGATTATATCCACTCGCAATGAGTTCACAAGGAATGCAGCTTTAGACAGTTGCTGGGACTTAAGGAAAAGAAAGGATCTCTGTAAAAGGAGCTTGTTTGCATCTTCCAGCATACAACGATGCAGGGAGCTGTTGTCACCTTTGAAACAGGAAATAATTCCTCAGCAGACCCTGACTGTAGCAAATCCTCTCCCCAGAGCTGTACTCTGAGATGGACTAAGACAGCCACTGTGTACATCTTCGACTTCACTTCCTCTAATTCCCCCTGGGTATTGGTCCTCCTCGCTTTAAATGCTGTAAGAAGCCTGATCTCTCAATCTTCAAAACTGTTCTTTCAGACATATCCCCAGAGTTCCATCTGAAATGCAAATTACATAAAAGGAATCCTATCGTCAAGCCTTTTAATGCTTATCCACTGTTTATTTTGAACATAACCTTTCCTTTTACGTACAGCATTTCTCTAGCTTGTCATTGACTATTTTTTCAACTGCTGCTGGACTTCATGATGCATGTTATAGATTCACCTTCCTGTTTGTGCTTCAGTTTCACTTGCCCAGAGAGCAATGTCTCTTTTTCATTATAAGCCCGTCATGCCTCAGTAGCAACATCTGTGCTGCAATCACTGTTGAAAGTGTTTCTCTGAACTGTGGGTTTGTAGACAGCAGTCCTTAGTCTGTTTATGCCCTTGTCATTTAGCTAATTCTTTAGCCCAGTGTACACTCATAATAATAACATTTGTCCTTTGGGTTGAACGAAAAGCATTTGAACCCTTTCAAATGATGAACATAAACTTATAAATACGTTCTGGGATTTAATAATTTATGTAAGTTTGGTAAATGGGAAAGAAGAAAAGAAAATGTAAATATTTTAGTAAGACCAGAATATGGTTGTATGAAGTTTTTACATATTTGAGCTGTGTTGTGTTAGTCTCTAAGGGCTGGTTCTATAGATGGGAATGGCCATGCTTGGTGACTTGAAATCATCATTGATTTCTGTGTCCTTCTTTCTGTGTATCTGTCTTTCTCTTTCATTTTCTCATACCCTCCTTCCCTCCCTCCCTCCCTCCCTCCCTCCCTCCCTCCCTCCCC-3’ |
| --- |

1. The co-expression genes of LNC_000280

| Target genes of LNC_000280 | Proliferation associated gene | Differentiation associated gene |
| --- | --- | --- |
| Ociad2 | √ |  |
| Ppp1r3c | √ |  |
| Agl | √ |  |
| Prima1 | √ |  |
| Shmt1 | √ |  |
| Mylk4 | √ |  |
| Slc25a25 | √ |  |
| Agt | √ | √ |
| Ppm1l | √ | √ |
| Idh2 | √ | √ |
| Gpt2 | √ | √ |
| Fgf1 | √ | √ |
| Klhl31 | √ | √ |
| Sorbs2 | √ | √ |
| Arhgap26 | √ | √ |
| Aqp4 | √ | √ |
| Ankrd9 | √ | √ |
| Camk2a |  | √ |
| Cntnap2 |  | √ |
| Phka1 |  |  |
| Nos1 |  |  |
| Rnf150 |  |  |
| Flt1 |  |  |
| Tango6 |  |  |
| Gaa |  |  |
| Plin4 |  |  |
| Phkg1 |  |  |
| Mfn1 |  |  |
| Mettl21e |  |  |
| Sod2 |  |  |
| Acss1 |  |  |
| Nfil3 |  |  |
| Maf |  |  |
| Maob |  |  |
| Ache |  |  |
| Ramp1 |  |  |
| Lrrfip1 |  |  |
| Pogk |  |  |
| Lrrc52 |  |  |
| Cmbl |  |  |
| Ldhb |  |  |
| Slc2a4 |  |  |
| Slc25a4 |  |  |
| Lpl |  |  |
| Acadm |  |  |
| Ppara |  |  |
| Abcb4 |  |  |
| Gpd2 |  |  |
| Sesn1 |  |  |
| Gadd45g |  |  |
| Ganc |  |  |
| Tpi1 |  |  |
| Esrrg |  |  |
| Eltd1 |  |  |
| Efcab6 |  |  |
| Riiad1 |  |  |
| D430041D05Rik |  |  |
| Cd36 |  |  |
| Map2k6 |  |  |
| Asb18 |  |  |
| Neurl2 |  |  |
| Fsd2 |  |  |
| Klhl33 |  |  |
| Mogat1 |  |  |
| Asb17 |  |  |
| Asb11 |  |  |
| Nfasc |  |  |
| Tiam1 |  |  |
| Ufsp1 |  |  |
| Obscn |  |  |
| Pptc7 |  |  |
| Asb15 |  |  |
| Ank3 |  |  |
| Nt5c1a |  |  |
| Ampd1 |  |  |
